# Clinically Suspected Atypical Pantothenate Kinase-Associated Neurodegeneration Without the Eye-of-the-Tiger Sign: A Case Report

**DOI:** 10.7759/cureus.113136

**Published:** 2026-07-22

**Authors:** Rasika Rode, Preetam Salunkhe, Jayshree Madavi, Pratiksha Virdande

**Affiliations:** 1 Department of Medicine, Mahatma Gandhi Institute of Medical Sciences, Wardha, IND; 2 Department of General Medicine, All India Institute of Medical Sciences (AIIMS), Nagpur, IND

**Keywords:** extrapyramidal syndrome, iron deposition, nbia, pantothenate kinase-associated neurodegeneration, pkan, susceptibility-weighted imaging

## Abstract

Pantothenate kinase-associated neurodegeneration (PKAN) is the most common form of neurodegeneration with brain iron accumulation (NBIA), typically characterized by extrapyramidal symptoms and the classical *eye-of-the-tiger *sign on MRI. However, atypical forms may lack this hallmark feature, leading to diagnostic challenges.

We report a 27-year-old male with a 12-year history of progressive tremors, gait disturbance, dysarthria, dysphagia, and behavioral changes. Neurological examination revealed extrapyramidal features, including dystonia and sialorrhea. MRI brain demonstrated bilateral T2 hypointensity in the globus pallidus with prominent blooming on susceptibility-weighted imaging (SWI), suggestive of iron deposition, without the classical eye-of-the-tiger sign. A diagnosis of clinically suspected atypical PKAN was made based on the characteristic clinical and radiological findings, and genetic confirmation by PANK2 mutation testing was advised.

Atypical PKAN often presents later with slower progression and may lack characteristic MRI findings. This case highlights the diagnostic limitations of conventional MRI and emphasizes the importance of SWI in detecting basal ganglia iron deposition.

PKAN should be considered in young patients with progressive extrapyramidal symptoms even in the absence of the eye-of-the-tiger sign. SWI can improve diagnostic accuracy and help reduce delays in diagnosis.

## Introduction

Pantothenate kinase-associated neurodegeneration (PKAN) is the most common subtype of neurodegeneration with brain iron accumulation (NBIA), a heterogeneous group of inherited progressive neurodegenerative disorders characterized by excessive iron deposition predominantly within the globus pallidus and substantia nigra [1,2]. PKAN is caused by pathogenic variants in the PANK2 gene, which encodes pantothenate kinase 2, a mitochondrial enzyme that catalyzes the first regulatory step in coenzyme A biosynthesis. Impaired enzyme function results in mitochondrial dysfunction, oxidative stress, neuronal degeneration, and abnormal iron accumulation within the basal ganglia, although the precise mechanisms responsible for selective iron deposition remain incompletely understood [1,3].

Clinically, PKAN is broadly classified into classic and atypical forms. Classic PKAN typically presents during early childhood with rapidly progressive dystonia, rigidity, pigmentary retinopathy, and early loss of ambulation. In contrast, atypical PKAN usually manifests later in adolescence or adulthood and follows a more slowly progressive and heterogeneous clinical course characterized by dystonia, tremor, Parkinsonism, dysarthria, psychiatric manifestations, and cognitive impairment [1,2]. This clinical variability frequently contributes to delayed diagnosis and may lead to confusion with other movement disorders or alternative NBIA syndromes.

Magnetic resonance imaging (MRI) is central to the evaluation of suspected PKAN. The characteristic *eye-of-the-tiger *sign, consisting of central T2 hyperintensity surrounded by hypointensity within the globus pallidus, has traditionally been regarded as a radiological hallmark of the disease [4]. However, this sign is not universally present and may be absent in early disease or in atypical phenotypes, thereby reducing its diagnostic sensitivity [4-6]. Advanced MRI techniques such as susceptibility-weighted imaging (SWI) are considerably more sensitive for detecting iron deposition and may reveal pathological basal ganglia changes even when conventional MRI findings are inconclusive [6,7].

We report a patient with clinically suspected atypical PKAN who presented with a slowly progressive extrapyramidal syndrome and bilateral globus pallidus iron deposition demonstrated on SWI despite the absence of the classical eye-of-the-tiger sign. This case highlights the diagnostic challenges associated with atypical PKAN and emphasizes the complementary role of SWI in facilitating the clinical recognition of NBIA disorders when characteristic conventional MRI findings are absent.

## Case presentation

A 27-year-old male presented with a 12-year history of gradually progressive neurological symptoms. His illness began at approximately 14 years of age with tremors involving both lower limbs, which later progressed to involve the upper limbs. By around 18 years of age, he developed an unsteady, broad-based gait with frequent falls. Over the next few years, he developed progressive dysarthria and dysphagia. His speech became slow and slurred, although comprehension was preserved. Subsequently, he developed oromandibular dystonia, sialorrhea, blepharospasm, impaired gaze fixation, and behavioral changes in the form of social disinhibition. By the time of presentation, he had become dependent on activities of daily living.

There was no history of similar illness in the family and no history of consanguinity. On examination, the patient was conscious and oriented, with a Glasgow Coma Scale score of 15/15.

Cranial nerve examination revealed bulbar dysfunction involving the lower cranial nerves. The patient had dysarthric speech, dysphagia, an absent gag reflex suggestive of glossopharyngeal and vagus nerve involvement, and tongue weakness consistent with hypoglossal nerve involvement. Motor examination revealed mildly increased tone in all four limbs, with preserved power of 5/5. Deep tendon reflexes were normal and symmetrical, and plantar responses were flexor bilaterally. Extrapyramidal features included bilateral resting tremors, dystonia, oromandibular dystonia, blepharospasm, dysarthria, and sialorrhea.

Baseline laboratory investigations are summarized in Table 1. Hematological evaluation showed mild anemia with a hemoglobin level of 10.98 g/dL. Peripheral smear examination revealed microcytic hypochromic red blood cells. Total leukocyte count and platelet count were within normal limits. Liver function parameters, including total and direct bilirubin, were within normal limits. Serum ceruloplasmin was within the laboratory reference range, making Wilson disease less likely; however, Wilson disease could not be definitively excluded in the absence of complete copper metabolism studies and ophthalmological documentation of Kayser-Fleischer rings.

**Table 1:** Laboratory investigations.

Investigation	Result	Reference range
Hemoglobin (g/dL)	10.98	13.0-17.0
Total leukocyte count (×10³/µL)	6.01	4.0-11.0
Platelet count (×10³/µL)	281	150-450
Total bilirubin (mg/dL)	0.22	0.2-1.2
Direct bilirubin (mg/dL)	0.09	0.0-0.3
Serum ceruloplasmin (g/L)	0.31*	0.20-0.60
Peripheral smear	Microcytic hypochromic red blood cells	Normocytic normochromic RBC morphology

Investigations 

**Figure 1:**
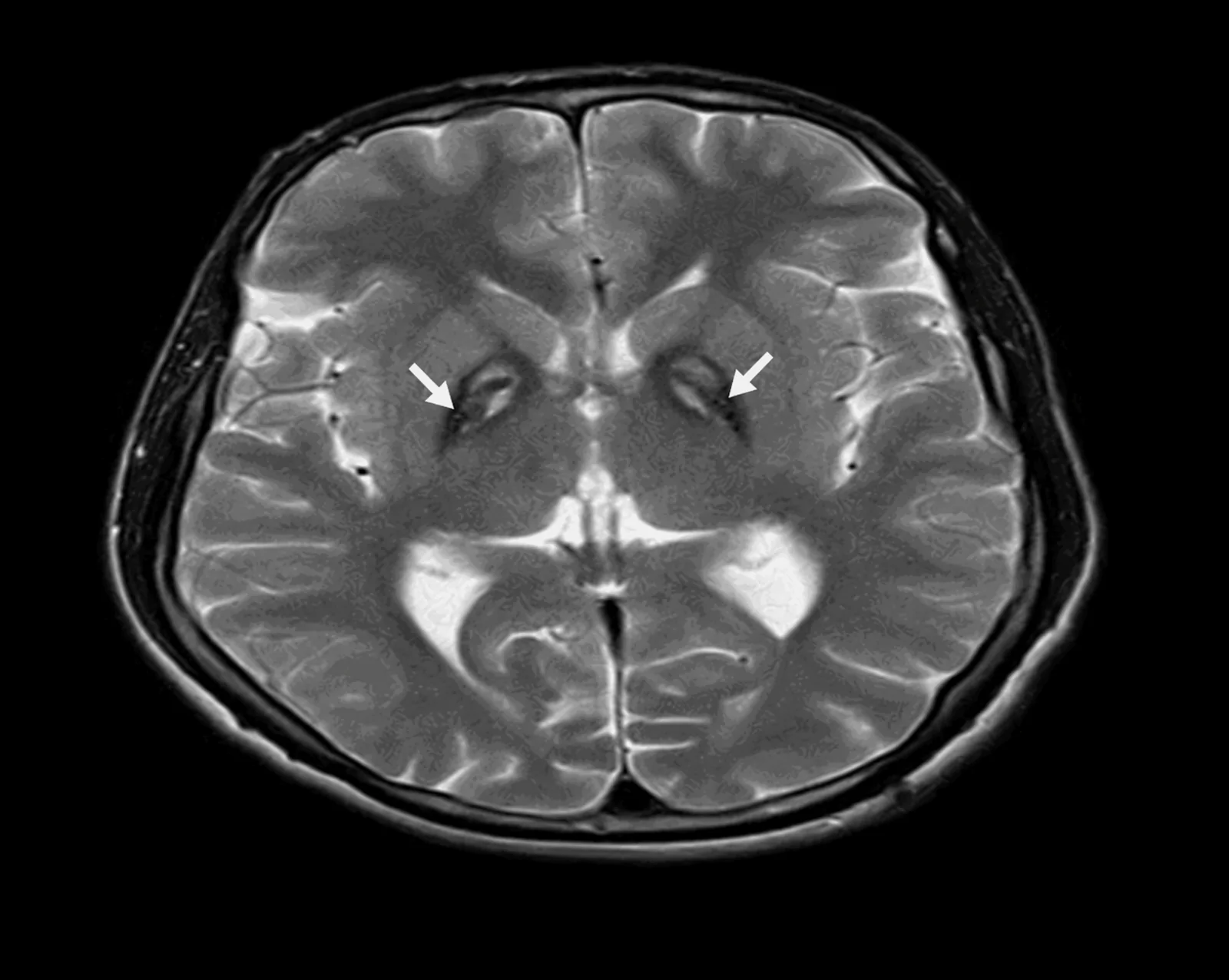
Axial T2-weighted MRI of the brain. Axial T2-weighted magnetic resonance imaging (MRI) of the brain demonstrates bilaterally symmetrical hypointensity in the medial globus pallidus (white arrows), suggestive of iron deposition. Notably, there is no central hyperintensity, and the classic *eye-of-the-tiger *sign is absent, indicating an atypical radiological presentation of pantothenate kinase-associated neurodegeneration (PKAN).

**Figure 2:**
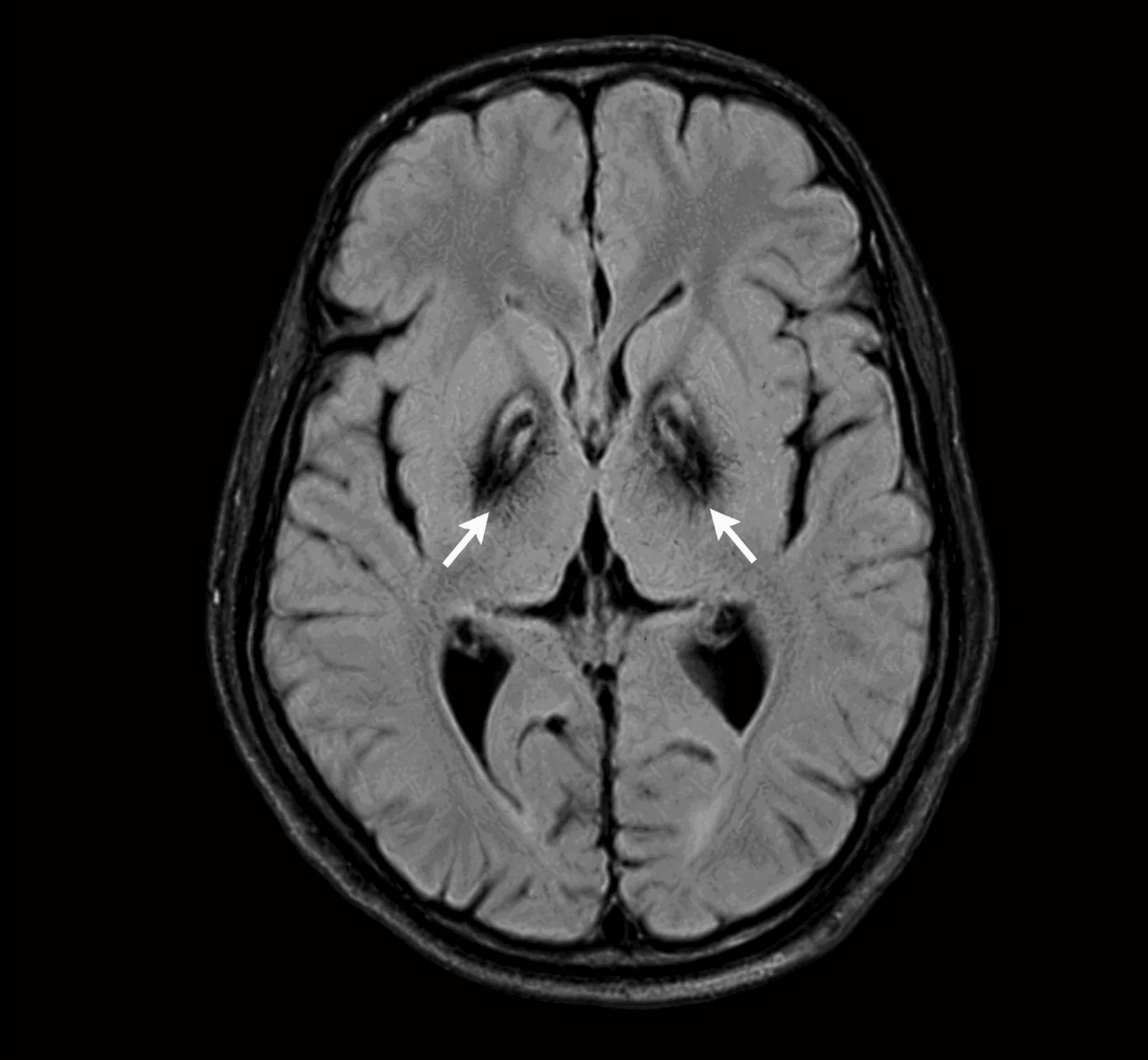
Axial susceptibility-weighted imaging (SWI) demonstrating prominent blooming artifacts in the bilateral globus pallidus (white arrows), consistent with iron accumulation. These findings support basal ganglia iron deposition and highlight the utility of SWI in detecting pathological changes in PKAN, particularly in the absence of classic T2-weighted MRI findings. PKAN, pantothenate kinase-associated neurodegeneration

**Figure 3:**
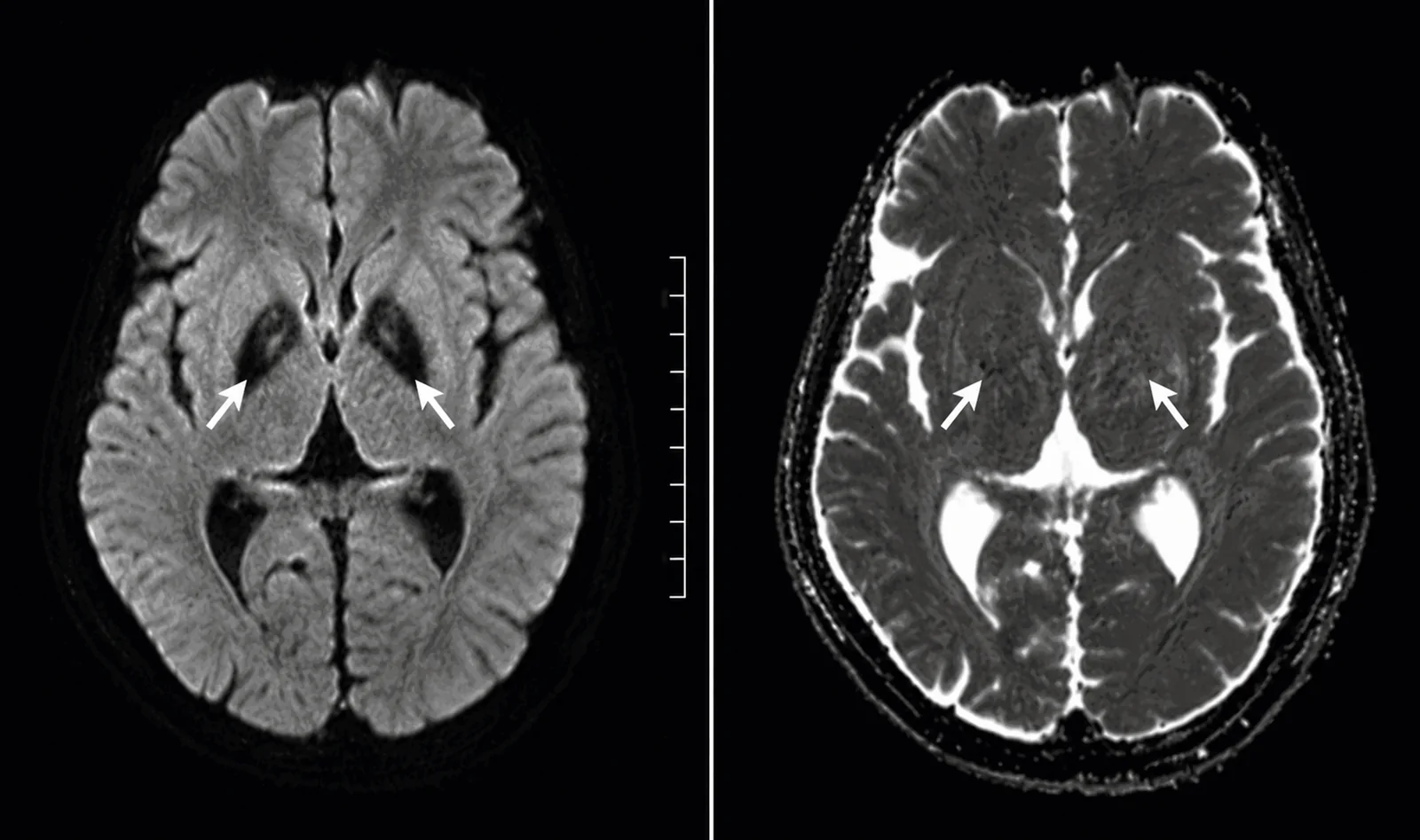
Axial diffusion-weighted imaging (DWI) and corresponding apparent diffusion coefficient (ADC) maps show the bilateral globus pallidus (white arrows) with no evidence of diffusion restriction, effectively excluding acute ischemic pathology.

Magnetic resonance imaging of the brain demonstrated bilaterally symmetrical T2 hypointensity involving the medial globus pallidus, suggestive of abnormal iron deposition. Notably, there was no central hyperintensity, and the classical *eye-of-the-tiger *sign was absent. SWI demonstrated prominent bilateral blooming within the globus pallidus, further supporting iron accumulation. Diffusion-weighted imaging with corresponding apparent diffusion coefficient maps showed no diffusion restriction, excluding acute ischemic pathology (Figures 1-3).

Baseline laboratory investigations are summarized in Table 1. Hematological evaluation showed mild anemia with a hemoglobin level of 10.98 g/dL, while leukocyte and platelet counts were within normal limits. Peripheral smear examination revealed microcytic hypochromic red blood cells. Liver function parameters, including total and direct bilirubin, were within normal limits. Serum ceruloplasmin was normal, making Wilson disease less likely.

Differential diagnosis

The differential diagnosis included other causes of progressive extrapyramidal neurodegeneration with basal ganglia involvement. Wilson's disease was considered because of the patient’s young age and movement disorder phenotype. However, the absence of hepatic manifestations, serum ceruloplasmin within the reference range, and neuroimaging findings favoring iron deposition made Wilson disease less likely, although it could not be definitively excluded without complete copper metabolism studies.

Other neurodegeneration with brain iron accumulation subtypes were also considered. However, based on the adolescent onset, slowly progressive course, extrapyramidal manifestations, bulbar dysfunction, behavioral changes, cognitive impairment, and selective globus pallidus iron deposition, the overall clinical-radiological phenotype was most suggestive of clinically suspected atypical pantothenate kinase-associated neurodegeneration. Definitive exclusion of other neurodegeneration with brain iron accumulation subtypes was limited by the absence of molecular genetic testing. Genetic confirmation with PANK2 mutation testing was therefore advised.

Treatment and follow-up 

The patient was managed symptomatically with pharmacological therapy directed toward control of extrapyramidal manifestations, including dystonia and associated motor symptoms. Supportive management included nutritional optimization, speech and swallowing rehabilitation, aspiration precautions, physiotherapy, and caregiver counselling regarding disease progression and supportive care needs. Because the diagnosis remained clinically presumptive, genetic testing for the PANK2 mutation was recommended for confirmation. The patient continues to remain under neurological follow-up for symptomatic management and monitoring of disease progression.

## Discussion

PKAN is one of the most common subtypes of NBIA, a heterogeneous group of inherited progressive neurodegenerative disorders characterized by pathological iron deposition, predominantly within the basal ganglia [1,2]. PKAN results from pathogenic variants in the *PANK2 *gene, which encodes pantothenate kinase 2, a mitochondrial enzyme involved in coenzyme A biosynthesis. Disruption of this pathway contributes to mitochondrial dysfunction, oxidative stress, progressive neuronal injury, and abnormal iron accumulation, although the precise mechanism linking PANK2 dysfunction to selective basal ganglia iron deposition remains incompletely understood [1,3].

Clinically, PKAN is broadly categorized into classic and atypical forms. Classic PKAN usually presents in early childhood with rapid progression, whereas atypical PKAN tends to manifest later in adolescence or adulthood and follows a slower, more heterogeneous clinical course [1,2]. Patients with atypical disease may present with dystonia, tremor, gait impairment, dysarthria, psychiatric manifestations, Parkinsonian features, or cognitive impairment, often creating diagnostic uncertainty [1,2]. The present case demonstrated several features consistent with atypical PKAN, including onset during adolescence, slowly progressive extrapyramidal symptoms over more than a decade, dysarthria, dysphagia, behavioral changes, cognitive impairment, and progressive functional decline. The delayed presentation and absence of dramatic childhood deterioration favored an atypical phenotype.

The classical radiological hallmark of PKAN is the *eye-of-the-tiger* sign on T2-weighted MRI, characterized by central hyperintensity within the globus pallidus surrounded by hypointense signal due to iron deposition [1,4]. Although this sign is strongly suggestive of PKAN, it is not universally present. Previous reports have shown that the eye-of-the-tiger sign may be absent in early disease, may evolve over time, or may be absent in selected patients with PANK2-related disease [4,5]. Reliance solely on this conventional MRI appearance may therefore delay diagnosis in clinically compatible cases.

Our case highlights an important diagnostic pitfall. Reliance on the classical eye-of-the-tiger sign alone may delay recognition of atypical PKAN in patients with otherwise characteristic clinical features. In our patient, SWI demonstrated bilateral pallidal iron deposition despite the absence of the classical T2-weighted appearance, emphasizing the complementary role of SWI in the evaluation of patients with clinically suspected NBIA.

Consistent with this observation, conventional MRI in our patient demonstrated bilateral globus pallidus hypointensity but lacked the characteristic central hyperintensity of the eye-of-the-tiger sign. However, SWI demonstrated bilateral pallidal blooming, supporting pathological iron deposition. SWI is highly sensitive to paramagnetic substances such as iron and can improve the detection of basal ganglia iron accumulation in NBIA disorders [6,7]. These findings suggest that SWI may provide valuable complementary evidence of basal ganglia iron deposition and facilitate earlier clinical recognition in patients with clinically suspected NBIA when conventional MRI findings are inconclusive. However, quantitative iron imaging was unavailable. Quantitative susceptibility mapping (QSM), R2* mapping, or region-of-interest signal intensity analysis would have provided a more objective assessment of iron burden, and the absence of these measurements is a limitation of this report [6,7].

Another limitation is the absence of serial neuroimaging over the patient’s 12-year clinical course. The patient presented to our center for the first time after a prolonged history of progressive neurological symptoms, and previous MRI studies or imaging records were not available. Financial constraints and delayed access to specialized neurological care may have contributed to the absence of earlier diagnostic evaluation. Longitudinal imaging would have provided additional information regarding the temporal evolution of iron deposition and disease progression.

The differential diagnosis included other NBIA subtypes, as these disorders may share overlapping extrapyramidal, cognitive, psychiatric, and radiological features [2,8]. Other NBIA subtypes were considered less likely on clinical and radiological grounds. PLA2G6-associated neurodegeneration (PLAN) was less likely because the patient did not have infantile-onset neuroregression, early hypotonia, prominent cerebellar ataxia, optic atrophy, or rapid early progression. Mitochondrial membrane protein-associated neurodegeneration (MPAN) was considered but was less favored because optic atrophy, prominent spasticity, and lower motor neuron features were not dominant manifestations. Beta-propeller protein-associated neurodegeneration (BPAN) was less likely in the absence of childhood-onset global developmental delay, seizures, severe expressive language impairment, and later abrupt Parkinsonian-dementia phenotype. Fatty acid hydroxylase-associated neurodegeneration (FAHN) was less likely because there was no prominent cerebellar ataxia, optic atrophy, or early spastic tetraparesis. Kufor-Rakeb syndrome was considered less likely because juvenile Parkinsonism, supranuclear gaze palsy, facial-faucial-finger myoclonus, and oculogyric crises were not characteristic findings in this case. Neuroferritinopathy was less favored because of the absence of a typical adult-onset choreiform or orofacial dystonia-dominant phenotype. Aceruloplasminemia was considered less likely because there were no systemic features such as diabetes mellitus, retinal degeneration, or biochemical evidence of systemic iron overload [2,8,9].

Nevertheless, because PANK2 genetic testing could not be performed, these alternative NBIA subtypes cannot be definitively excluded. Therefore, the diagnosis has been presented as clinically suspected atypical PKAN rather than genetically confirmed PKAN. This limitation is important because molecular confirmation remains the definitive method for establishing the PKAN subtype in patients with overlapping clinical and radiological features [1,2].

Pharmacological approaches may include anticholinergic agents, baclofen, benzodiazepines, botulinum toxin, and other therapies aimed at controlling dystonia and motor symptoms [1,9]. Deep brain stimulation may provide benefit in carefully selected patients with severe disabling dystonia, although response varies [10]. Iron chelation strategies such as deferiprone have also been explored, but evidence for consistent clinical benefit remains limited [11].

## Conclusions

Atypical PKAN may present without the classical eye-of-the-tiger sign, leading to significant diagnostic delays. Clinicians should consider NBIA disorders, including clinically suspected atypical PKAN, in young patients presenting with progressive extrapyramidal syndromes despite the absence of characteristic conventional MRI findings. SWI provides valuable evidence of basal ganglia iron deposition and may facilitate earlier clinical recognition. Nevertheless, molecular confirmation remains the diagnostic gold standard whenever feasible.
